# Unique Prenatal Diagnosis of a Coronary Sinus Aneurysm

**DOI:** 10.1016/j.jaccas.2026.107822

**Published:** 2026-04-09

**Authors:** Zareh Torabyan, Christopher Lindblade, Michael Nguyen, Ryan Johnson

**Affiliations:** Department of Pediatric Cardiology, Phoenix Children's Hospital, Phoenix, Arizona, USA

**Keywords:** computed tomography, congenital heart defect, echocardiography

## Abstract

**Background:**

Congenital cardiac aneurysms are very rare in pediatric and fetal populations and can compose a range of anomalies, including ventricular, atrial, and coronary sinus aneurysm. Accurate recognition is essential because embryologic origins, imaging features, and clinical implications significantly differ.

**Case Summary:**

A 16-year-old primigravida young woman was evaluated at 23 5/7 weeks’ gestation in the fetal cardiology clinic for evaluation of a suspected thoracic cyst. Fetal echocardiography and magnetic resonance imaging demonstrated a large aneurysm arising from dilated coronary sinus with impressive posterior and inferior extension without persistent left superior vena cava. No hydrops, arrhythmia, or pericardial effusion were present. Postnatal echocardiography and computed tomography angiography confirmed a rare presentation of a coronary sinus aneurysm.

**Discussion:**

Coronary sinus aneurysms arise from abnormal venous wall development or hemodynamic stress and must be distinguished from ventricular or atrial outpouchings, which carry variable risks of rupture, arrhythmia, or hydrops. Early fetal diagnosis permits accurate classification, anticipatory counseling, and tailored postnatal management.

**Take-Home Messages:**

This case illustrates the importance of distinguishing coronary sinus aneurysm from ventricular or atrial aneurysms. Prenatal recognition facilitates accurate diagnosis, risk assessment, counseling, and delivery planning.

## Visual Summary

**Figure:**
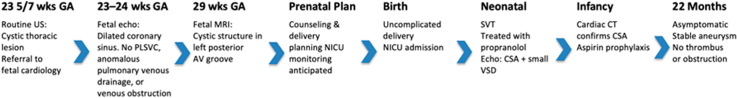

## History of Presentation

**Figure 1:**
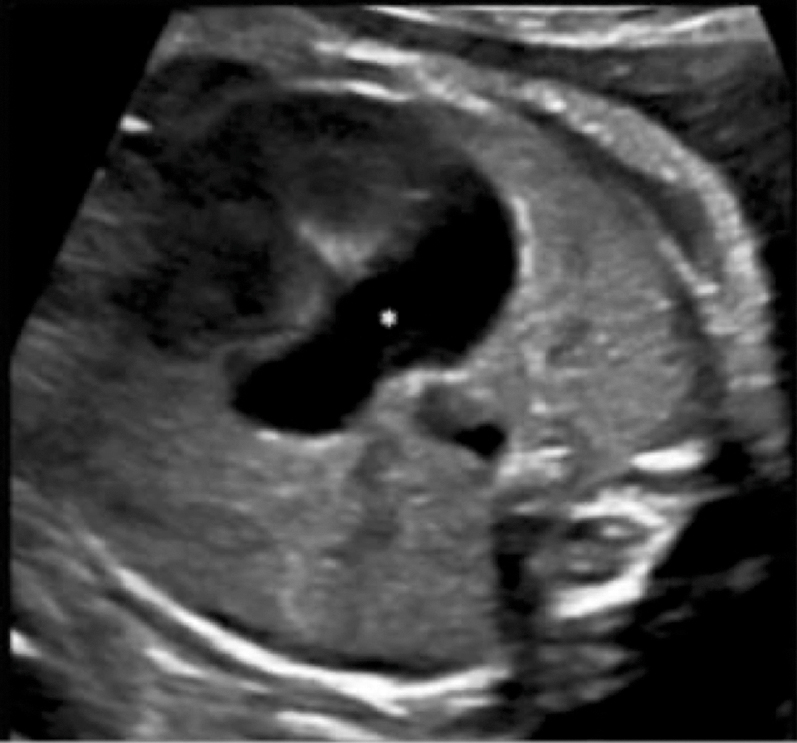
Fetal Echocardiography Demonstrating Enlarged Structure Along the Left Posterior Atrioventricular Groove Measuring 1.5 × 2.2 cm (Asterisk) Inferior sweeps localize the structure to the region of the coronary sinus.

A 16-year-old primigravida young woman was referred at 23 5/7 weeks’ gestation to the fetal cardiology clinic for evaluation of a suspected cystic thoracic lesion identified on routine prenatal ultrasound. Fetal echocardiography excluded congenital diaphragmatic hernia and revealed a markedly dilated coronary sinus. The right atrium and ventricle were mildly enlarged with normal biventricular systolic function. No persistent left superior vena cava was identified. There were normal connections and drainage of the ductus venosus and mesenteric and hepatic veins. Intracardiac and segmental anatomy were otherwise normal, including normal pulmonary venous return. There was no evidence of pericardial effusion, arrhythmia, or hydrops. Interestingly, the aneurysm had significant posterior and inferior extension (Figure 1, Video 1).

**Take-Home Message**

• It is important to recognize that not every cystic cardiac structure reflects myocardial disease and recognizing the underlying embryologic and hemodynamic distinctions is key to providing clear counseling and appropriate treatment.

## Past Medical History

There was no known maternal medical history, fetal extracardiac anomalies, or family history of congenital heart disease. Prenatal course was otherwise uncomplicated before referral.

## Differential Diagnosis

Given the prenatal appearance of a cystic cardiac structure, the differential diagnosis included ventricular aneurysm or diverticulum, atrial appendage aneurysm, coronary sinus aneurysm (CSA), and other cystic thoracic lesions.

**Visual Summary:**
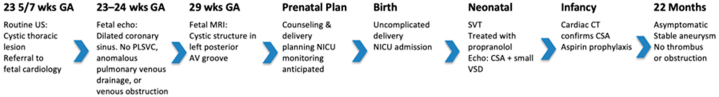
Clinical Timeline of Prenatal Diagnosis and Postnatal Course of CSA The timeline outlines key prenatal imaging findings, postnatal arrhythmia, and subsequent imaging confirming the diagnosis with stable follow-up. AV = atrioventricular; CSA = coronary sinus aneurysm; CT = computed tomography; GA = gestational age; MRI = magnetic resonance imaging; NICU = neonatal intensive care unit; PLSVC = persistent left superior vena cava; SVT = supraventricular tachycardia; US = ultrasound; VSD = ventricular septal defect.

## Investigations

**Figure 2:**
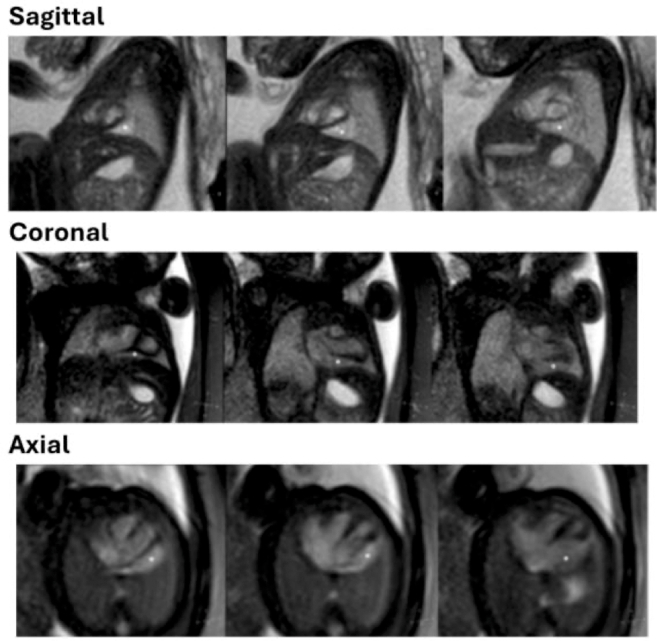
Fetal Magnetic Resonance Imaging at 29 Weeks Demonstrating a Well-Circumscribed Cystic Structure (Asterisk) Along the Left Posterior Atrioventricular Groove Extending Inferiorly Along the Thoracic Surface of the Diaphragm on Sagittal, Coronal, and Axial Sequences

A fetal magnetic resonance imaging was performed at 29 weeks’ gestation. Sagittal, coronal, and axial sequences showed a well-defined cystic structure along the left posterior atrioventricular groove (Figure 2), consistent with marked dilation of the coronary sinus. No extracardiac extension, mass effect, or associated thoracic abnormalities were identified.

**Figure 3:**
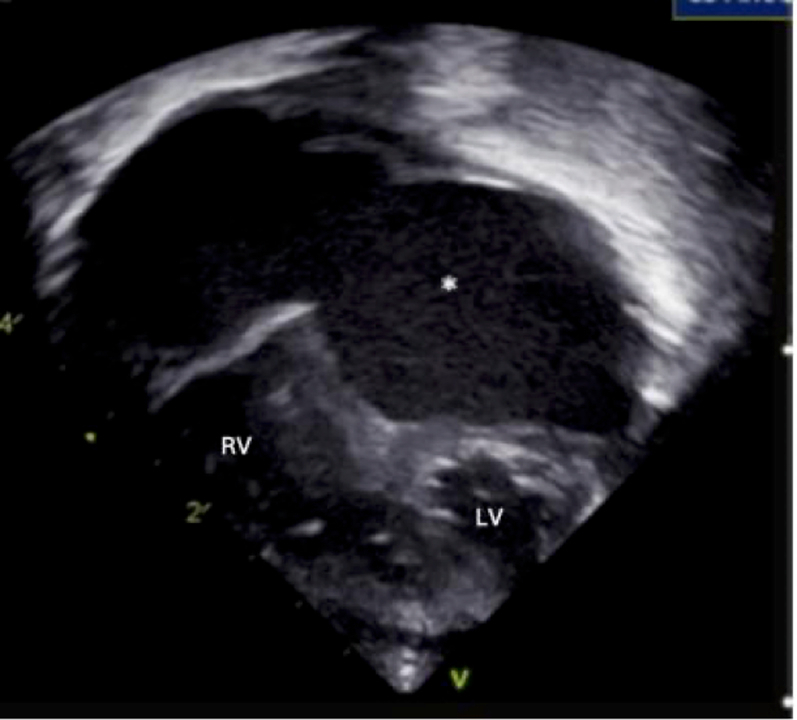
Postnatal Transthoracic Echocardiogram Showing Large Coronary Sinus Aneurysm (Asterisk) Along the Left Posterior Atrioventricular Groove LV = left ventricle; RV = right ventricle.

**Figure 4:**
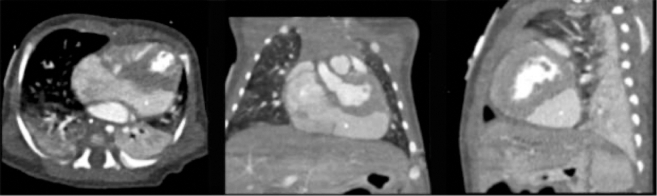
Postnatal Cardiac Computed Tomography Angiography Depicting Enlarged Coronary Sinus (Asterisk) Along the Left Posterior Atrioventricular Groove on Axial, Coronal, and Sagittal Images

**Figure 5:**
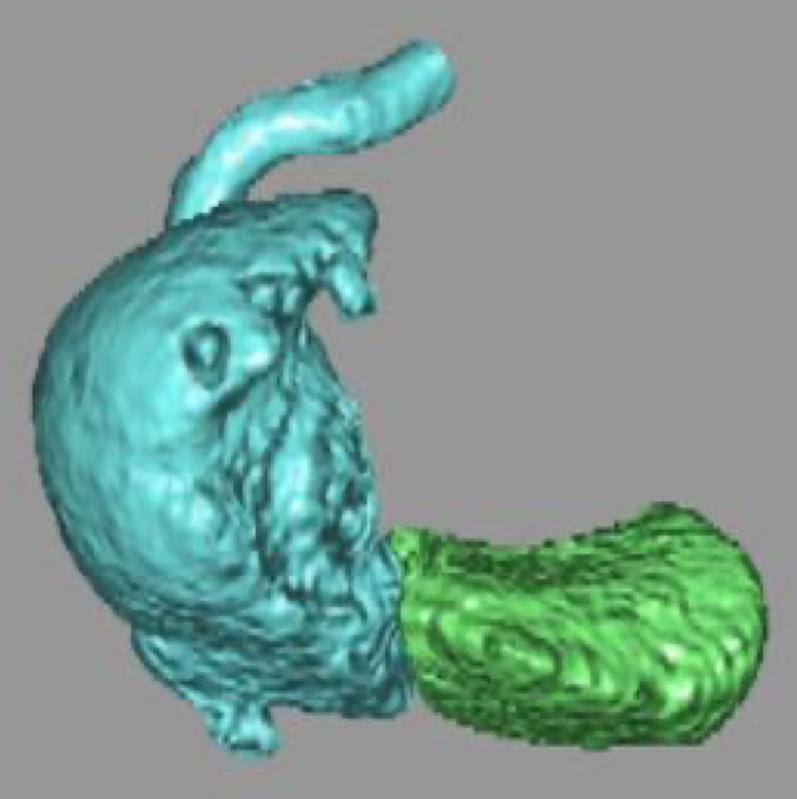
Postnatal 3-Dimensional Cardiac Computed Tomography Displaying Coronary Sinus Aneurysm (Green) and Dilated Right Atrium (Blue)

Postnatal transthoracic echocardiography confirmed a large, extensive CSA and a small perimembranous ventricular septal defect. In addition, the echocardiogram confirmed normal pulmonary venous drainage, no evidence of persistent left superior vena cava, and no evidence of coronary sinus ostial stenosis or atresia (Figure 3, Video 2). Cardiac computed tomography angiography was performed to delineate the extent of the aneurysm (Figure 4). Axial, coronal, and sagittal views confirmed marked enlargement of the coronary sinus without evidence of venous anomalies or coronary artery fistulae. 3-dimensional reconstruction (Figure 5, Video 3) demonstrated posterior extension along the left atrioventricular groove over the superior surface of the diaphragm. There was no compression of the pulmonary veins or mitral valve annulus on postnatal imaging.

## Management

The family was counseled that management during pregnancy would focus on close monitoring and coordinated delivery planning. Although hemodynamic instability was not anticipated, the infant would require neonatal intensive care unit admission for rhythm and hemodynamic monitoring, including repeat echocardiography and advanced cardiac imaging to better define the lesion. Potential management pathways were discussed, including observation, antiarrhythmic therapy, and initiation of anticoagulation due to thromboembolic risk.

The infant was delivered at term without complications. In the neonatal period, the patient developed supraventricular tachycardia (concealed accessory pathway, atrioventricular reentry tachycardia). The supraventricular tachycardia was successfully managed with propranolol, which was discontinued at 1 year of age without recurrence. The patient was managed conservatively with aspirin for thromboembolism prophylaxis.

## Outcome and Follow-Up

The patient has remained clinically stable. At 22 months of age, the patient has not developed labored breathing, diaphoresis, or pallor and reaching normal growth and developmental milestones. Serial imaging demonstrates stable aneurysmal dilation of the coronary sinus without thrombus formation, obstruction to venous return, or cardiac dysfunction.

## Discussion

The coronary sinus develops from the left horn of the sinus venosus which drains to the right atrium during early cardiac development. When this segment fails to regress normally, or when it is exposed to elevated pressure or volume load, dilation can occur, producing an aneurysm.^1^ These aneurysms may occur from intrinsic wall weakness, venous hypertension, or fistulous communication between a coronary artery or sinus of Valsalva and the coronary sinus.^2^, ^3^, ^4^ On the other hand, ventricular outpouchings, including aneurysms and diverticula, are myocardial in origin. Ventricular aneurysms have a broad neck, asynchronous contractility, and may progress in size, whereas ventricular diverticula have a narrow neck with synchronous contractility with reports of rupture in the literature.^5^^,^^6^ Furthermore, congenital atrial aneurysms develop from congenital atrial wall dysplasia or absent pericardium and are often associated with progressive enlargement, increased thromboembolic risk, and atrial arrhythmias.^7^

Echocardiography is the primary imaging tool for identifying and differentiating these lesions. A CSA appears as a thin-walled structure along the posterior left atrioventricular groove, showing venous-type to-fro flow into the right atrium.^1^ In contrast, ventricular aneurysms and diverticula typically arise from the left ventricular apex, and less commonly the left ventricular free wall.^6^^,^^8^ Atrial aneurysms, which are very rare and mostly acquired, may arise from the free wall or atrial appendage and display contractile activity.^7^

In this case, fetal echocardiography revealed a markedly enlarged, noncontractile coronary sinus with normal systemic and pulmonary venous drainage and no persistent left superior vena cava, which is a common association with coronary sinus dilation.^9^ These findings supported the diagnosis of an isolated CSA rather than secondary enlargement.

On axial, coronal, and sagittal computed tomography views (Figure 4), the coronary sinus appeared markedly enlarged and confirmed the absence of venous anomalies or coronary artery fistulae. The 3-dimensional reconstruction (Figure 5, Video 3) helped visualize how the aneurysm extended posteriorly along the left atrioventricular groove along the superior surface of the diaphragm. This case of a CSA is associated with a small perimembranous ventricular septal defect and neonatal supraventricular tachycardia.

Prior published cases suggested that CSAs are seen in the setting of other associated lesions such as coronary artery fistulas or venous anomalies have led to catheter or surgical interventions.^2^, ^3^, ^4^^,^^9^ There are limited data on the course of isolated coronary artery aneurysms; however, the benign clinical course observed in our patient can add to the growing evidence that isolated cases may follow a favorable course. In a review of 3,216 postmortem congenital heart disease cases described by Van Praagh,^10^ only 10 patients (0.31%) had aneurysms of the left and right horns of the sinus venosus, with 6 (66.7%) of these cases involving the left sinus horn (embryologic origin of the coronary sinus), demonstrating the scarcity of this lesion. These were consistently associated with complex congenital heart disease, often with atrioventricular valve abnormalities such as tricuspid and mitral atresia. One-half of the CSAs communicated with a ventricular chamber, mostly the left ventricle. One patient was seen to also have Wolff-Parkinson-White syndrome.^10^ This is important because CSAs can be easily overlooked or mistaken for other cardiac outpouchings in the presence of these findings.

Ventricular and atrial aneurysms generally carry greater morbidity. In a large prenatal series, Shuplock et al^6^ reported a 17% mortality rate among fetuses with ventricular outpouchings, primarily due to hydrops and severe ventricular dysfunction. Atrial appendage aneurysms are clinically important because of their tendency to cause atrial dysrhythmias and systemic embolization.^7^

## Conclusions

Prenatal identification of CSAs allows for accurate classification, assessment of potential risks, and appropriate prenatal counseling and delivery planning. Management should be conservative, focusing on rhythm monitoring and serial imaging to assess progression in size or development of thrombus.

## Funding Support and Author Disclosures

The authors have reported that they have no relationships relevant to the contents of this paper to disclose.

## Appendix

Video 1 Fetal Echocardiography Demonstrating Enlarged Structure Along the Left Posterior Atrioventricular Groove Measuring 1.5 × 2.2 cm (Asterisk) Inferior sweeps localize the structure to the region of the coronary sinus.

Video 2 Post-Natal Transthoracic Echocardiogram Showing Large Coronary Sinus Aneurysm (Asterisk) Along the Left Posterior Atrioventricular Groove

Video 3 Post-Natal 3-Dimensional Cardiac Computed Tomography Displaying Coronary Sinus Aneurysm (Green) and Dilated Right Atrium (Blue)
